# LUMEN: Prototype Conversational AI to Streamline Dementia Assessments

**DOI:** 10.1192/bjo.2025.10185

**Published:** 2025-06-20

**Authors:** Song Ling Tang, Alexander Robertson, Huizhi Liang, John-Paul Taylor, Judith Harrison

**Affiliations:** ^1^Hertfordshire Partnership University NHS Foundation Trust, Hertfordshire, United Kingdom; ^2^Newcastle University, Newcastle, United Kingdom

## Abstract

**Aims:** Dementia assessments are time-intensive and often distressing for patients and caregivers. Underdiagnosis of non-Alzheimer’s disease subtypes remains prevalent. This study aimed to develop and evaluate LUMEN (Large Language Model for Understanding and Monitoring Elderly Neurocognition), a prototype conversational AI to automate caregiver-collateral data collection before clinical appointments. Our goals were to reduce clinician time per assessment, improve diagnostic accuracy across dementia subtypes, and standardise caregiver assessments.

**Methods:** LUMEN’s development integrated a Patient, Public, and Professional Involvement (PPPI) process, incorporating stakeholder workshops, a modified Delphi process with 130 clinicians, and iterative consultations to identify key diagnostic priorities, such as functional impairments, safety concerns, and inclusivity. Four open-source 7B-parameter large language models (LLMs) – Mistral, Llama2, Zephyr, and Phi2 – were evaluated for efficiency (token count), readability (Flesch Reading Ease), and contextual relevance (cosine similarity to clinical dialogues). Mistral:7B was selected and fine-tuned using automated hyperparameter adjustments (GridSearchCV), advanced prompt engineering (chain-of-thought, flipped classroom techniques), and BLEU-scored linguistic refinement. A prototype interface was tested using 16 clinician-simulated caregiver dialogues derived from case vignettes spanning dementia subtypes and normal cognition. LUMEN’s diagnostic outputs were compared with clinician-derived diagnoses using the Area Under the Receiver Operating Characteristic (AUROC) curve and agreement measured via Cohen’s kappa. Usability was assessed via the System Usability Scale (SUS).

**Results:** LUMEN demonstrated strong performance in distinguishing dementia from normal cognition (AUROC=0.89) but moderate subtype differentiation (AUROC=0.66). Agreement between LUMEN and clinician evaluations was substantial (Cohen’s κ=0.82). However, Lewy body dementia (DLB) identification lagged due to symptom-reporting inaccuracies. System Usability Scale (SUS) scores (mean=82/100) exceeded the ‘excellent’ threshold (≥80). PPPI feedback highlighted LUMEN’s potential to standardise assessment and reduce waiting times.

**Conclusion:** LUMEN is a promising conversational AI tool for improving dementia diagnostics. Gathering caregivers’ collateral input before appointments could streamline workflows within existing outpatient systems and improve clinical accuracy. Real-world trials would help assess workflow integration and mitigate vignette-based biases from simulated testing, such as the overrepresentation of typical phenotypes.

This study was conducted in collaboration with Mr Bede Burston, Dr Elizabeth Robertson, and Dr Donncha Mullin, whose contributions were invaluable.

